# Challenges to Evaluating Respiratory Syncytial Virus Mortality in Bangladesh, 2004–2008

**DOI:** 10.1371/journal.pone.0053857

**Published:** 2013-01-24

**Authors:** Lauren J. Stockman, W. Abdullah Brooks, Peter K. Streatfield, Mustafizur Rahman, Doli Goswami, Kamrun Nahar, Mohammed Z. Rahman, Stephen P. Luby, Larry J. Anderson

**Affiliations:** 1 National Center for Immunization and Respiratory Diseases, Centers for Disease Control and Prevention. Atlanta, Georgia, United States of America; 2 International Center for Diarrheal Disease Research-Bangladesh, Dhaka, Bangladesh; National Institutes of Health, United States of America

## Abstract

**Background:**

Acute lower respiratory illness is the most common cause of death among children, globally. Data are not available to make accurate estimates on the global mortality from respiratory syncytial virus (RSV), specifically.

**Methods:**

Respiratory samples collected from children under 5 years of age during 2004 to 2008 as part of population-based respiratory disease surveillance in an urban community in Dhaka, Bangladesh were tested for RSV, human metapneumovirus (HMPV), human parainfluenza virus (PIV) types 1, 2, and 3, influenza and adenovirus by RT-PCR. Verbal autopsy data were used to identify children who died from respiratory illness in a nearby rural community. Significance of the correlation between detections and community respiratory deaths was determined using Spearman's coefficient.

**Results:**

RSV activity occurred during defined periods lasting approximately three months but with no clear seasonal pattern. There was no significant correlation between respiratory deaths and detection of any of the respiratory viruses studied.

**Conclusion:**

Outbreaks of respiratory viruses may not be associated with deaths in children in the study site; however, the few respiratory deaths observed and community-to-community variation in the timing of outbreaks may have obscured an association. An accurate assessment of respiratory virus-associated deaths will require detections and death data to come from the same location and a larger study population.

## Introduction

Acute lower respiratory illness (ALRI), specifically pneumonia, is the most common cause of death among children, globally [1]. Over 90% of pneumonia deaths occur in 40 countries primarily in Asia and Africa, and two-thirds of these occur in only ten, including Bangladesh [2]. Many pneumonia-related deaths occur outside of hospitals, resulting in underestimation from hospital-based studies [3]. An estimate of agent-specific mortality cannot be directly determined for most community deaths as they occur in settings where diagnostic testing cannot be performed and the relationship between deaths in the hospital and those in the community are uncertain [4], [5]. Consequently, estimates of agent-specific respiratory deaths, including those associated with respiratory syncytial virus (RSV), based on extrapolations from mortality in hospitalized children are of uncertain reliability [1]. The best and most recent estimate suggests that RSV causes up to 200,000 ALRI deaths in young children each year [6]. However, that study noted that a more accurate estimate of global RSV-associated mortality requires more population-based surveillance data, and these estimates, in turn, are important for prioritizing global RSV research and prevention resources.

Diagnostic specimens are rarely available from children who die of respiratory deaths in the community. In the United States, mortality associated with RSV has been estimated by identifying temporal patterns in respiratory virus detections and deaths [7], and using national viral surveillance data in statistical models [8]. This approach has worked well in settings where there are good quality data on timing of RSV detections and respiratory deaths. A cohort study of 256 newborns in Bangladesh reported RSV as a seasonal, predominant pathogen resulting in pneumonia hospitalization [9] but the proportion of respiratory deaths associated with RSV detections is unknown. The International Centre for Diarrheal Disease Research, Bangladesh (icddr,b) has population-based surveillance in a community in urban Dhaka for acute respiratory illness where RSV infection is confirmed by molecular laboratory testing. In addition, as part of longstanding demographic and health surveillance in a rural community 50 kilometers outside of Dhaka, verbal autopsy information is regularly collected. In this report, we describe our efforts to apply an approach using temporal patterns of RSV and other respiratory virus detections at the urban surveillance site combined with deaths from a nearby site to estimate the proportion of respiratory deaths associated with RSV and other respiratory virus infections in Bangladesh.

## Methods

### Specimen data

Nasopharyngeal washes (NPWs) from children under 5 years of age were collected during 2004 to 2008 as part of population-based respiratory disease surveillance in Kamalapur, a low-income community in the urban capital city of Dhaka [10]. Briefly, field workers made weekly visits to approximately 5,000 households under active surveillance and referred children with signs of illness to a clinic. Nasopharyngeal washes were collected from every fifth child from the surveillance area who had an acute infectious respiratory illness, as previously described [10], [11].

In the laboratory, specimens were processed and tested for respiratory viruses using TaqMan real-time reverse transcriptase-polymerase chain reaction (rRT-PCR) with primers and probes specific for RSV as described previously [12] and for human metapneumovirus (HMPV), human parainfluenza virus (PIV) types 1, 2, and 3 and adenovirus (protocols available from CDC upon request). A sample was considered positive if a characteristic RT-PCR curve was observed with a desired cycle threshold (CT<39). Influenza virus was detected by tissue culture, described elsewhere [13], as part of an earlier study of influenza in this population [10].

### Verbal autopsy data

Deaths of children under 5 years of age during 2004 to 2008 were identified by a demographic and health surveillance program. Deaths were recorded and investigated in Matlab, a rural community approximately 50 km south of Dhaka, where approximately 225,000 people live within the surveillance area. These households were visited every two months to ascertain birth and death information. Verbal autopsy (VA) has been widely used as a method to determine cause of death in places where deaths occur without medical supervision. A VA questionnaire was used by trained community health workers to interview the caretakers of the deceased and assign medical causes of death based on signs and symptoms of illness which preceded death [14], [15]. Death associated with respiratory illness was defined by the following codes for underlying cause of death (ICD-10): Influenza and pneumonia (J11–J18); other acute lower respiratory infection (J20–J22); acute upper respiratory infections (J00–J06), other diseases of the respiratory tract (J30–J39) and chronic lower respiratory disease (J40–J47)

### Analysis

The Spearman non-parametric correlation was used to assess whether the number of respiratory deaths in the Matlab community increased when the frequency of detections for individual respiratory viruses increased in Dhaka. Each respiratory virus was analyzed separately pairing the percent of detections positive with the number of respiratory deaths for each study month and converting the values into ranks for correlation using SAS version 9.1. The correlation coefficient (r) ranges from −1 to +1 with 0 indicating no correlation. The correlation was considered statistically significant if p< = 0.05 and the association was positive.

The Institutional Review Board at the International Center for Diarrheal Disease Research, Bangladesh, approved this study. Written informed consent was obtained from the primary adult care-taker for all children from whom samples were collected as part of population-based respiratory disease surveillance in Kamalapur.

## Results

### Seasonality of viral detections

**Figure 1:**
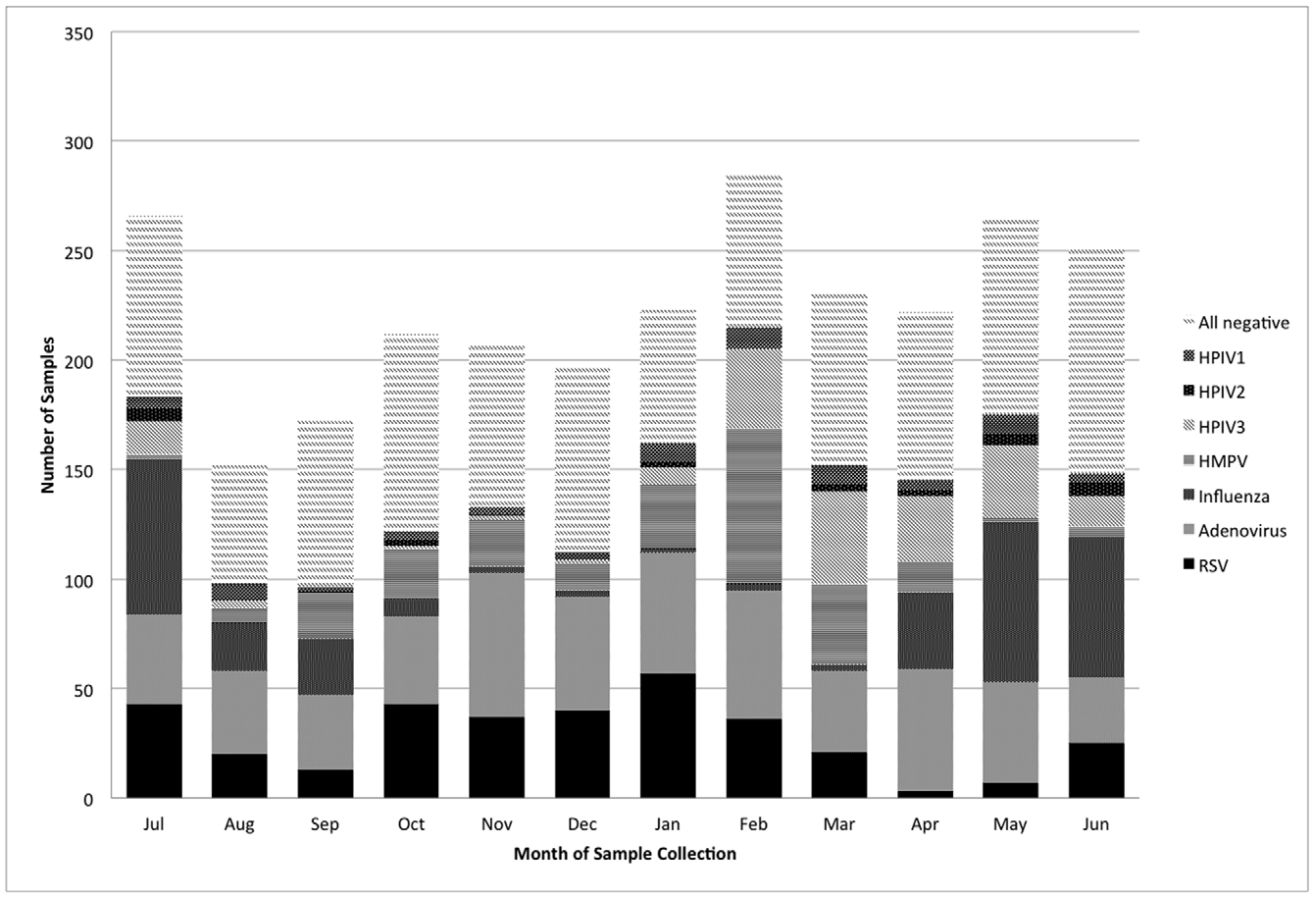
Nasopharyngeal wash samples collected and laboratory detections, by month, in children under 5 years with acute lower respiratory infection, Dhaka, 2004—2008.

2,401 nasopharyngeal wash samples collected from 2,266 children under 5 years old with respiratory illness between April 4, 2004 and February 28, 2008 were available for analysis from Kamalapur in Dhaka. All samples were tested and 1,458 (61%) were positive for at least one virus. Adenovirus was the most common, identified in 23% of samples, followed by RSV (14%), influenza (13%), HMPV (10%), PIV-3 (8%), PIV-1 (3%) and PIV-2 (1%), **Figure 1**. More than one virus was identified in 261 samples (11%) and the most common of these co-infections was RSV with adenovirus (26%).

**Figure 2:**
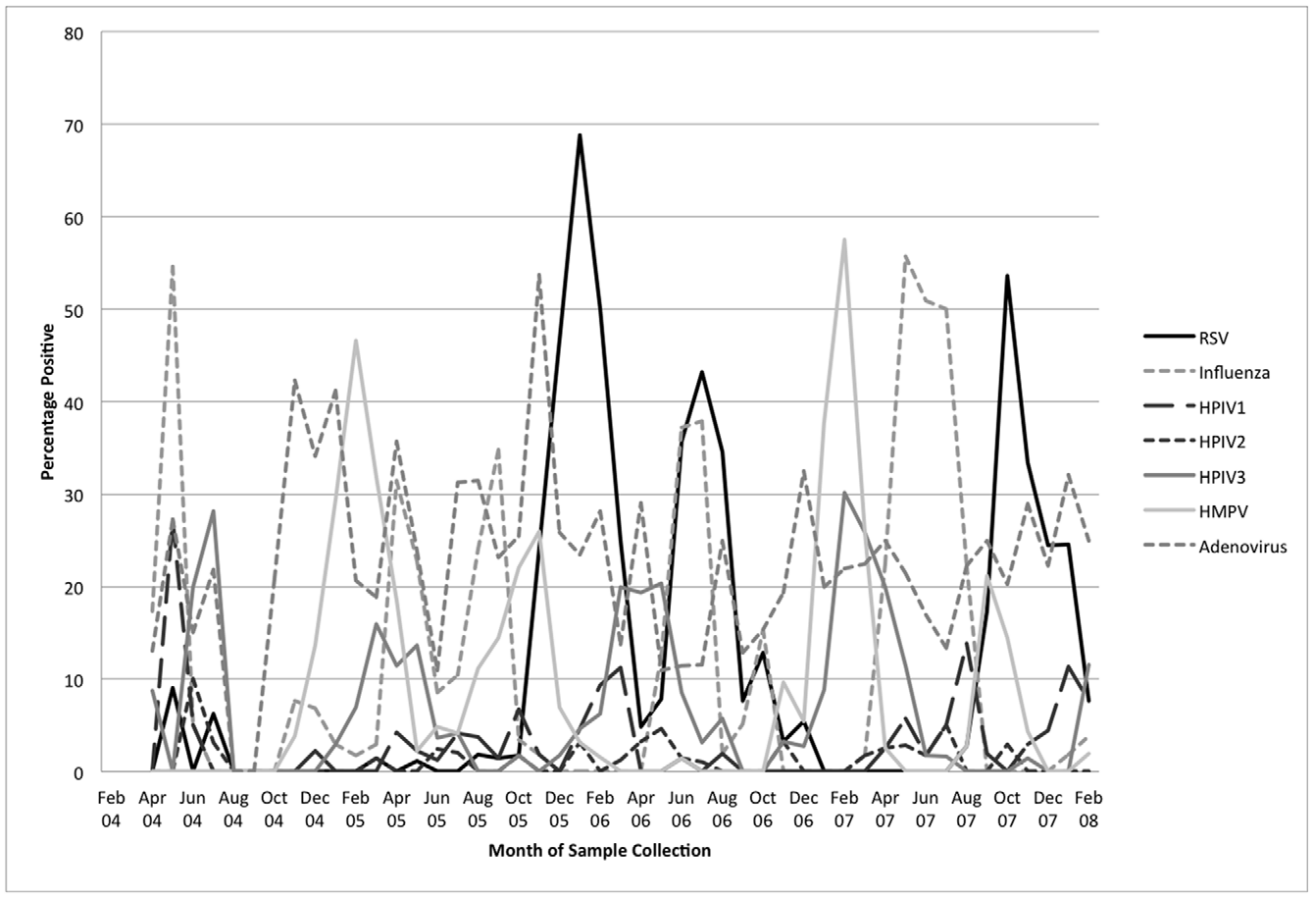
Laboratory detections, by month and year, in children under 5 years with acute lower respiratory infection, Dhaka, 2004—2008.

Annual data suggests RSV activity occurred during defined periods lasting approximately three months with no clear seasonal pattern. During the 4 study years there was one peak in January 2006, July 2006 and October 2007. There were at least 2 months with low RSV detections between peaks. During these study years, influenza, HMPV and PIV-3 activity showed more consistent seasonality with all viruses having a peak in each study year. Influenza virus peaks occurred in the second or third quarters of the year and PIV-3 during the end of the first quarter and into the second. HMPV had 4 episodes of peak activity in the first and fourth quarters of the year. Adenovirus was common and present year-round throughout the study period and there were too few PIV-1 and PIV-2 detections to identify seasonal patterns, **Figure 2**.

### Deaths due to respiratory illness

**Figure 3:**
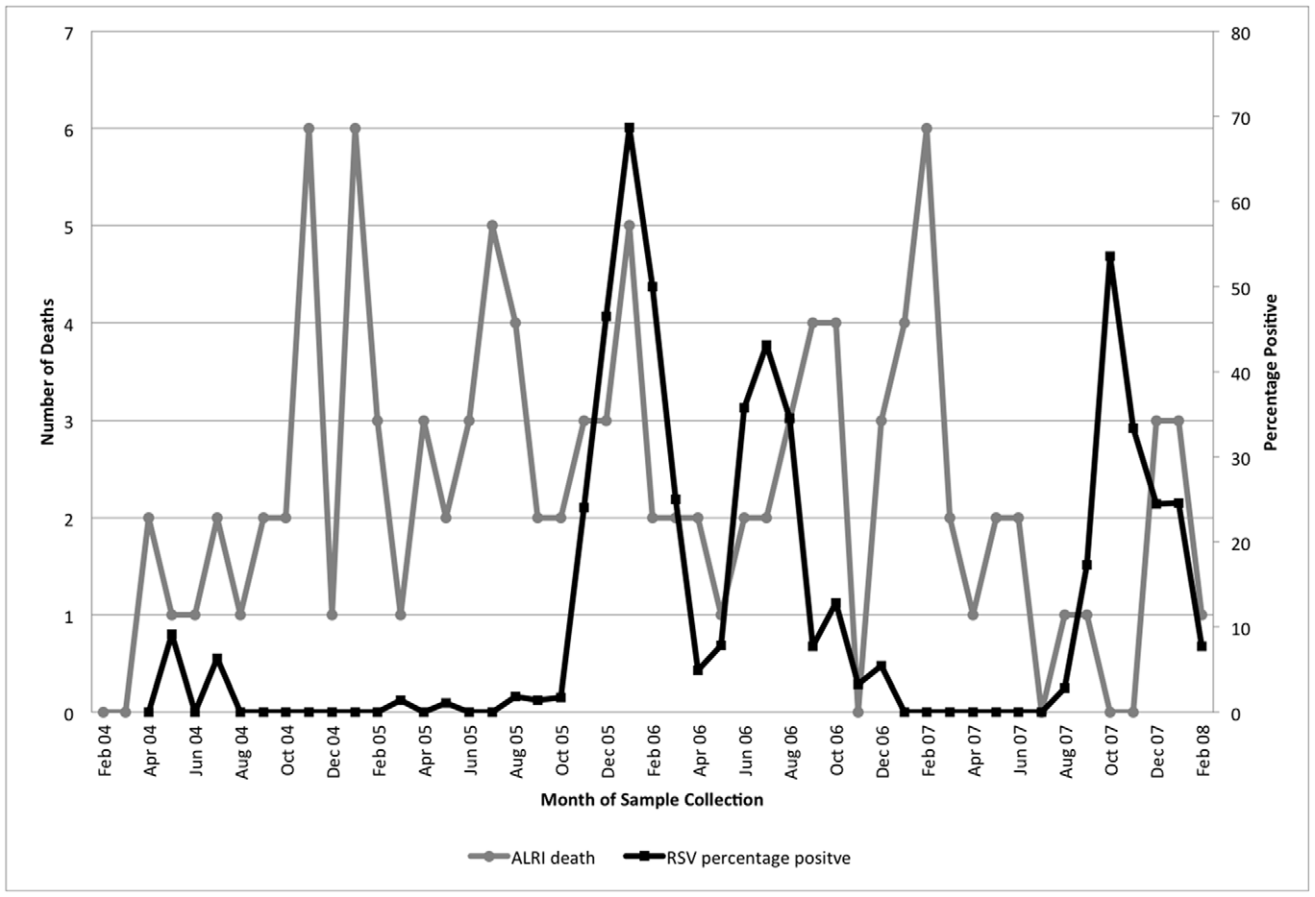
Deaths from acute lower respiratory illness, Matlab, and RSV detections, Dhaka, among children under 5 years, 2004—2008.

**Figure 4:**
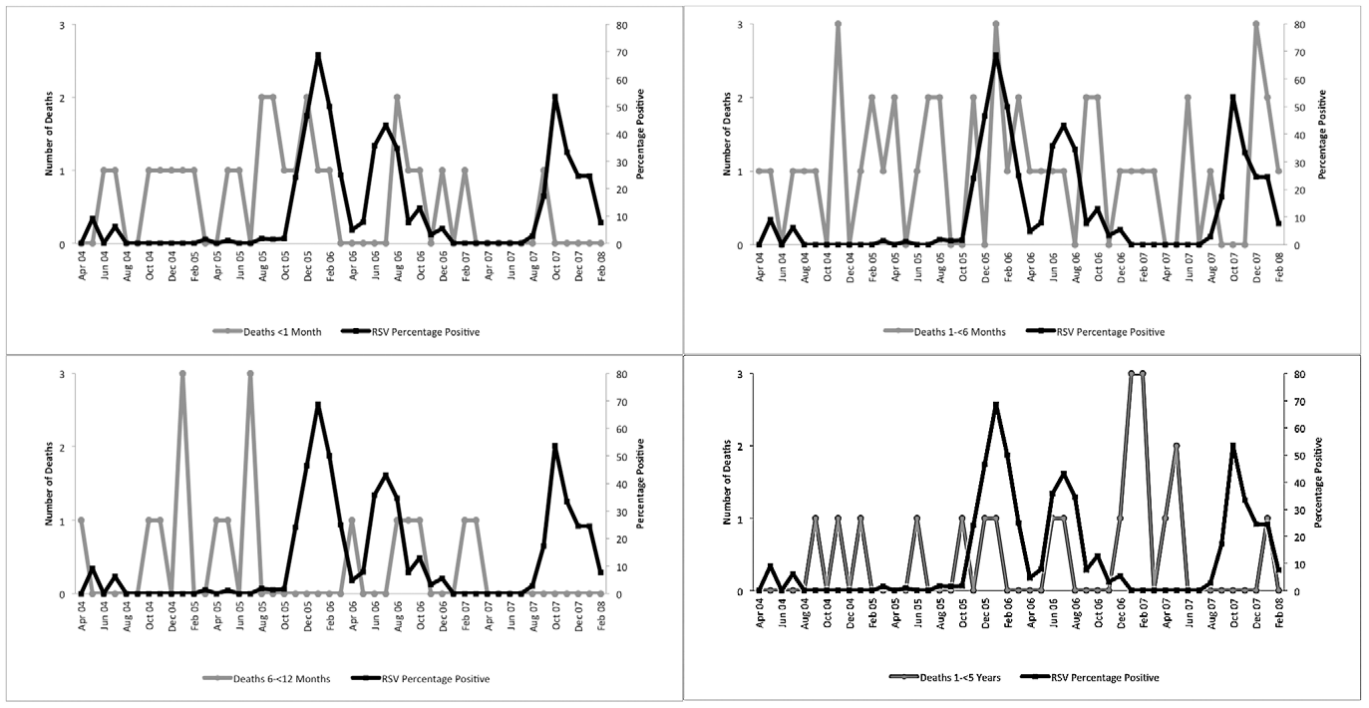
Deaths from acute lower respiratory illness, Matlab, and RSV detections, Dhaka, among children under 5 years, by age group, April 2004–February 2008.

Between April 4, 2004 and February 28, 2008, there were 111 deaths in rural Matlab due to respiratory illness in children under 5 years old, **Figure 3**. Of these, 108 (97%) were due to pneumonia from unspecified organism (ICD-10 J18), 2 (2%) due to asthma (ICD-10 J45) and 1 (1%) due to unspecified acute lower respiratory infection (ICD-10 J22). The average age at death was 6.7 months (range 0–59 months). Cumulatively, more deaths occurred in January than in other months but a January increase was not consistent throughout the study period. There was no apparent difference in the seasonality of death by age groups, **Figure 4**.

### Correlation of RSV and respiratory deaths

Cumulatively, the highest percentage of samples positive for RSV during the study period occurred in January. However, winter-time (December–February) RSV detection was not consistent across study years and RSV detections in Dhaka and deaths from respiratory illness in Matlab did not correlate with RSV detections year-to-year, r = −0.10, p = 0.52. No positive and significant correlation was evident for the other viruses: r = −0.10, p = 0.50 for influenza, r = 0.17, p = 0.25 for HMPV, r = 0.25, p = 0.09 for adenovirus, r = −0.22, p = 0.14 for PIV-1, r = −0.37, p = 0.01 for PIV-2 and r = −0.07, p = 0.65 for PIV-3.

## Discussion

RSV has been associated with seasonal mortality of young children in the US [7], Brazil [16] and Indonesia [4] and pneumonia deaths have been noted to correspond with the peak season of bronchiolitis in Bangladesh in 2003 [17]. Using four years of data, we found that although both RSV detections and respiratory deaths were relatively high in the winter months, the increase in RSV detections was not accompanied by an increase in death during individual years. Of the eight study months with four or more respiratory deaths, only one matched a peak in RSV detections. This could indicate that RSV does not impact respiratory deaths in the young child in Bangladesh. There are, however, other explanations for not finding a significant association between RSV and other respiratory virus outbreaks and respiratory deaths. The timing of RSV outbreaks in the community where specimens were tested may not have matched the timing of RSV outbreaks in the community where verbal autopsies were performed, there may have been a problem with the accuracy in classification of respiratory deaths, or the number of observed deaths was too few to accurately assess correlations with RSV or the other respiratory virus detections. The potential problems identified in this study suggest ways to improve future efforts to assess community mortality from respiratory virus infections.

First, it is essential that surveillance for respiratory virus circulation represent activity in the community where deaths are assessed. Surveillance data from the United States suggests that year-to-year variation in timing of RSV outbreaks in the same community as well as community-to-community variation in the same year [21], [22] and indicates the importance of using detection data from the same community where the mortality is assessed. Previous studies of RSV detections Bangladesh found overall peak activity during winter months in Dhaka [18], [19] while a rural community had peak activity during the monsoon season and not during winter months [9]. In our study, RSV detections showed well-defined increases in activity but the timing of these increases was too varied to infer longer-term patterns. There are differences in both population density and living conditions between urban Dhaka and Matlab, which may affect RSV transmission. Population density is an important contributor patterns of RSV season in the United States and has been found to affect duration and timing of seasons [20]. Of note, the incidence of clinical pneumonia in Matlab is less than half that in Dhaka [15]. Although these two sites are separated by less than 50 kilometers, variation in timing of local RSV outbreaks has been previously noted within short distances [21], [22] and the degree of variation may be greater in this setting. To be confident about linking RSV detections to health outcomes in a single community, data on RSV circulation from the same community are needed.

The small number of respiratory deaths identified by the verbal autopsies made it difficult to establish correlations in RSV activity with these deaths. Verbal autopsy has been validated, shown to be sensitive for respiratory death surveillance [14] and used previously in Bangladesh to provide cause of death data to policies and programs [23]. However, in this study, one or two more or fewer deaths would change the timing of peaks in deaths. This would make correlations of RSV detections with these peaks unstable and highly susceptible to random events that would affect a peak in the number of deaths. It is possible that respiratory deaths would lag behind an increase in viral detection, however we see no consistent increase in deaths 1 or 2 months after RSV detection.

Etiology studies have shown that RSV is predominantly associated with pneumonia and severe respiratory illness in infants [18], [19], [24], [25] and for this analysis, we assumed that deaths followed by respiratory illness in this age group could be attributed to RSV. However, overlap in respiratory virus seasons and co-infections complicate efforts to link detections to a single disease in the community. In this study, the timing of influenza and human metapneumovirus detections were distinct from that for RSV detections, suggesting that circulation of these viruses would not have affected determination of RSV deaths. The timing of adenovirus and parainfluenza virus detections did overlap with RSV detections for some months. Studies of circulation of respiratory viruses in some tropical climates have shown both overlap among these viruses and less consistent seasonal patterns of circulation than those in more temperate locations [26]. Thus, we cannot be sure if circulation patterns of any of the respiratory viruses observed in Dhaka were indicative of that in Matlab where deaths were assessed. It is important, however, to understand potential overlaps in circulation and studies looking at links between health outcomes and virus detections should include detection of the spectrum of potentially etiologic pathogens.

To accrue sufficient numbers of deaths for a more robust analysis of RSV-associated mortality in developing countries, studies will likely require several sites with each site using detections and death data come from the same location. Another approach that has been considered for determining RSV-associated mortality is a probe study that measures the reduction in mortality with use of an effective RSV-specific preventive measure. The only presently available preventive measure is immune prophylaxis. When available, an effective vaccine could also serve this purpose. Although our findings do not reliably inform RSV-associated mortality, they do identify factors that should be considered in the design of future studies of RSV disease burden in developing countries.
